# Supplementary material for: Facing a blind alley - Experiences of stress-related exhaustion: a qualitative study
Source: BMJ Open. 2020 Sep 2;10(9):e038230. doi: 10.1136/bmjopen-2020-038230 (PMC7470632; doi:10.1136/bmjopen-2020-038230)
Supplement: Author's manuscript [file bmjopen-2020-038230.draft_revisions.pdf]

BMJ Open is committed to open peer review. As part of this commitment we make the peer review history of every article we publish publicly available.

When an article is published we post the peer reviewers' comments and the authors' responses online. We also post the versions of the paper that were used during peer review. These are the versions that the peer review comments apply to.

The versions of the paper that follow are the versions that were submitted during the peer review process. They are not the versions of record or the final published versions. They should not be cited or distributed as the published version of this manuscript.

BMJ Open is an open access journal and the full, final, typeset and author-corrected version of record of the manuscript is available on our site with no access controls, subscription charges or pay-per-view fees (http://bmjopen.bmj.com).

If you have any questions on BMJ Open's open peer review process please email info.bmjopen@bmj.com

BMJ Open

Facing a blind alley
Experiences of stress-related exhaustion

Journal:	*BMJ Open*
Manuscript ID	bmjopen-2020-038230
Article Type:	Original research
Date Submitted by the Author:	03-Mar-2020

Complete List of Authors:	Alsén, Sara; University of Gothenburg Institute of Health and Care Sciences, Sahlgrenska Academy<br>Ali, Lilas; Health and Care Sciences, Sahlgrenska academy<br>Ekman, Inger; Institute of Health and Care Sciences, Sahlgrenska Academy, University of Gothenburg; University of Gothenburg Centre for Person-Centred Care, Sahlgrenska Academy, University of Gothenburg<br>Fors, Andreas; Institute of Health of Care Sciences, Sahlgrenska Academy, University of Gothenburg; Gothenburg Centre for Person-Centred Care (GPCC),

Keywords:	PRIMARY CARE, MENTAL HEALTH, QUALITATIVE RESEARCH

SCHOLARONE™
Manuscripts

I, the Submitting Author has the right to grant and does grant on behalf of all authors of the Work (as defined in the below author licence), an exclusive licence and/or a non-exclusive licence for contributions from authors who are: i) UK Crown employees; ii) where BMJ has agreed a CC-BY licence shall apply, and/or iii) in accordance with the terms applicable for US Federal Government officers or employees acting as part of their official duties; on a worldwide, perpetual, irrevocable, royalty-free basis to BMJ Publishing Group Ltd ("BMJ") its licensees and where the relevant Journal is co-owned by BMJ to the co-owners of the Journal, to publish the Work in this journal and any other BMJ products and to exploit all rights, as set out in our licence.

The Submitting Author accepts and understands that any supply made under these terms is made by BMJ to the Submitting Author unless you are acting as an employee on behalf of your employer or a postgraduate student of an affiliated institution which is paying any applicable article publishing charge ("APC") for Open Access articles. Where the Submitting Author wishes to make the Work available on an Open Access basis (and intends to pay the relevant APC), the terms of reuse of such Open Access shall be governed by a Creative Commons licence – details of these licences and which Creative Commons licence will apply to this Work are set out in our licence referred to above.

Other than as permitted in any relevant BMJ Author's Self Archiving Policies, I confirm this Work has not been accepted for publication elsewhere, is not being considered for publication elsewhere and does not duplicate material already published. I confirm all authors consent to publication of this Work and authorise the granting of this licence.

For peer review only

Reporting checklist for qualitative study.

Based on the SRQR guidelines.

Instructions to authors

Complete this checklist by entering the page numbers from your manuscript where readers will find each of the items listed below.

Your article may not currently address all the items on the checklist. Please modify your text to include the missing information. If you are certain that an item does not apply, please write "n/a" and provide a short explanation.

Upload your completed checklist as an extra file when you submit to a journal.

In your methods section, say that you used the SRQRreporting guidelines, and cite them as:

O'Brien BC, Harris IB, Beckman TJ, Reed DA, Cook DA. Standards for reporting qualitative research: a synthesis of recommendations. Acad Med. 2014;89(9):1245-1251.

		Reporting Item	Page Number
**Title**			
	#1	Concise description of the nature and topic of the study identifying the study as qualitative or indicating the approach (e.g. ethnography, grounded theory) or data collection methods (e.g. interview, focus group) is recommended	1

For peer review only

Abstract

	#2	Summary of the key elements of the study using the abstract format of the intended publication; typically includes background, purpose, methods, results and conclusions	1

Introduction

Problem formulation	#3	Description and signifcance of the problem / phenomenon studied: review of relevant theory and empirical work; problem statement	1,2
Purpose or research question	#4	Purpose of the study and specific objectives or questions	3

Methods

Qualitative approach and research paradigm	#5	Qualitative approach (e.g. ethnography, grounded theory, case study, phenomenolgy, narrative research) and guiding theory if appropriate; identifying the research paradigm (e.g. postpositivist, constructivist / interpretivist) is also recommended; rationale. The rationale should briefly discuss the justification for choosing that theory, approach, method or technique rather than other options available; the assumptions and limitations implicit in those choices and how those choices influence study conclusions and transferability.	3

For peer review only

		As appropriate the rationale for several items might be discussed together.	
Researcher characteristics and reflexivity	#6	Researchers' characteristics that may influence the research, including personal attributes, qualifications / experience, relationship with participants, assumptions and / or presuppositions; potential or actual interaction between researchers' characteristics and the research questions, approach, methods, results and / or transferability	11
Context	#7	Setting / site and salient contextual factors; rationale	3
Sampling strategy	#8	How and why research participants, documents, or events were selected; criteria for deciding when no further sampling was necessary (e.g. sampling saturation); rationale	3
Ethical issues pertaining to human subjects	#9	Documentation of approval by an appropriate ethics review board and participant consent, or explanation for lack thereof; other confidentiality and data security issues	3
Data collection methods	#10	Types of data collected; details of data collection procedures including (as appropriate) start and stop dates of data collection and analysis, iterative process, triangulation of sources / methods, and modification of procedures in response to evolving study findings; rationale	3

For peer review only

Data collection instruments and technologies	#11	Description of instruments (e.g. interview guides, questionnaires) and devices (e.g. audio recorders) used for data collection; if / how the instruments(s) changed over the course of the study	3
Units of study	#12	Number and relevant characteristics of participants, documents, or events included in the study; level of participation (could be reported in results)	3
Data processing	#13	Methods for processing data prior to and during analysis, including transcription, data entry, data management and security, verification of data integrity, data coding, and anonymisation / deidentification of excerpts	3,4
Data analysis	#14	Process by which inferences, themes, etc. were identified and developed, including the researchers involved in data analysis; usually references a specific paradigm or approach; rationale	4,5
Techniques to enhance trustworthiness	#15	Techniques to enhance trustworthiness and credibility of data analysis (e.g. member checking, audit trail, triangulation); rationale	3

Results/findings

Syntheses and interpretation	#16	Main findings (e.g. interpretations, inferences, and themes); might include development of a theory or model, or integration with prior research or theory	4-10

For peer review only

Links to empirical data	#17	Evidence (e.g. quotes, field notes, text excerpts, photographs) to substantiate analytic findings	4-8
**Discussion**			
Intergration with prior work, implications, transferability and contribution(s) to the field	#18	Short summary of main findings; explanation of how findings and conclusions connect to, support, elaborate on, or challenge conclusions of earlier scholarship; discussion of scope of application / generalizability; identification of unique contributions(s) to scholarship in a discipline or field	9-10
Limitations	#19	Trustworthiness and limitations of findings	11
**Other**			
Conflicts of interest	#20	Potential sources of influence of perceived influence on study conduct and conclusions; how these were managed	11
Funding	#21	Sources of funding and other support; role of funders in data collection, interpretation and reporting	11

The SRQR checklist is distributed with permission of Wolters Kluwer © 2014 by the Association of American Medical Colleges. This checklist was completed on 02. March 2020 using https://www.goodreports.org/, a tool made by the EQUATOR Network in collaboration with Penelope.ai

For peer review only

COREQ (COnsolidated criteria for REporting Qualitative research) Checklist

A checklist of items that should be included in reports of qualitative research. You must report the page number in your manuscript where you consider each of the items listed in this checklist. If you have not included this information, either revise your manuscript accordingly before submitting or note N/A.

Topic	Item No.	Guide Questions/Description	Reported on Page No.
**Domain 1: Research team and reflexivity**			
Personal characteristics			
Interviewer/facilitator	1	Which author/s conducted the interview or focus group?	11
Credentials	2	What were the researcher's credentials? E.g. PhD, MD	1
Occupation	3	What was their occupation at the time of the study?	1
Gender	4	Was the researcher male or female?	1
Experience and training	5	What experience or training did the researcher have?	1
Relationship with participants			
Relationship established	6	Was a relationship established prior to study commencement?	3
Participant knowledge of the interviewer	7	What did the participants know about the researcher? e.g. personal goals, reasons for doing the research	3
Interviewer characteristics	8	What characteristics were reported about the inter viewer/facilitator? e.g. Bias, assumptions, reasons and interests in the research topic	3
**Domain 2: Study design**			
Theoretical framework			
Methodological orientation and Theory	9	What methodological orientation was stated to underpin the study? e.g. grounded theory, discourse analysis, ethnography, phenomenology, content analysis	4
Participant selection			
Sampling	10	How were participants selected? e.g. purposive, convenience, consecutive, snowball	3
Method of approach	11	How were participants approached? e.g. face-to-face, telephone, mail, email	3
Sample size	12	How many participants were in the study?	3
Non-participation	13	How many people refused to participate or dropped out? Reasons?	3
Setting			
Setting of data collection	14	Where was the data collected? e.g. home, clinic, workplace	3
Presence of non-participants	15	Was anyone else present besides the participants and researchers?	3
Description of sample	16	What are the important characteristics of the sample? e.g. demographic data, date	3
Data collection			
Interview guide	17	Were questions, prompts, guides provided by the authors? Was it pilot tested?	3
Repeat interviews	18	Were repeat inter views carried out? If yes, how many?	3
Audio/visual recording	19	Did the research use audio or visual recording to collect the data?	3
Field notes	20	Were field notes made during and/or after the inter view or focus group?	3
Duration	21	What was the duration of the inter views or focus group?	3
Data saturation	22	Was data saturation discussed?	3
Transcripts returned	23	Were transcripts returned to participants for comment and/or	3

For peer review only

For peer review only - http://bmjopen.bmj.com/site/about/guidelines.xhtml

Topic	Item No.	Guide Questions/Description	Reported on Page No.
		correction?	
**Domain 3: analysis and findings**			
Data analysis			
Number of data coders	24	How many data coders coded the data?	4
Description of the coding tree	25	Did authors provide a description of the coding tree?	4
Derivation of themes	26	Were themes identified in advance or derived from the data?	4
Software	27	What software, if applicable, was used to manage the data?	4
Participant checking	28	Did participants provide feedback on the findings?	4
Reporting			
Quotations presented	29	Were participant quotations presented to illustrate the themes/findings? Was each quotation identified? e.g. participant number	5-9
Data and findings consistent	30	Was there consistency between the data presented and the findings?	9-10
Clarity of major themes	31	Were major themes clearly presented in the findings?	4-10
Clarity of minor themes	32	Is there a description of diverse cases or discussion of minor themes?	4-10

Developed from: Tong A, Sainsbury P, Craig J. Consolidated criteria for reporting qualitative research (COREQ): a 32-item checklist for interviews and focus groups. *International Journal for Quality in Health Care*. 2007. Volume 19, Number 6: pp. 349 – 357

Once you have completed this checklist, please save a copy and upload it as part of your submission. DO NOT include this checklist as part of the main manuscript document. It must be uploaded as a separate file.

For peer review only

BMJ Open

Facing a blind alley
Experiences of stress-related exhaustion – a qualitative interview study

Journal:	*BMJ Open*
Manuscript ID	bmjopen-2020-038230.R1
Article Type:	Original research
Date Submitted by the Author:	19-May-2020
Complete List of Authors:	Alsén, Sara; University of Gothenburg Institute of Health and Care Sciences, Sahlgrenska Academy<br>Ali, Lilas; Health and Care Sciences, Sahlgrenska academy<br>Ekman, Inger; Institute of Health and Care Sciences, Sahlgrenska Academy, University of Gothenburg; University of Gothenburg Centre for Person-Centred Care, Sahlgrenska Academy, University of Gothenburg<br>Fors, Andreas; Institute of Health of Care Sciences, Sahlgrenska Academy, University of Gothenburg; Gothenburg Centre for Person-Centred Care (GPCC),
**Primary Subject Heading**:	Mental health
Secondary Subject Heading:	Mental health, Qualitative research, Public health
Keywords:	PRIMARY CARE, MENTAL HEALTH, QUALITATIVE RESEARCH, PUBLIC HEALTH

SCHOLARONE™
Manuscripts

I, the Submitting Author has the right to grant and does grant on behalf of all authors of the Work (as defined in the below author licence), an exclusive licence and/or a non-exclusive licence for contributions from authors who are: i) UK Crown employees; ii) where BMJ has agreed a CC-BY licence shall apply, and/or iii) in accordance with the terms applicable for US Federal Government officers or employees acting as part of their official duties; on a worldwide, perpetual, irrevocable, royalty-free basis to BMJ Publishing Group Ltd ("BMJ") its licensees and where the relevant Journal is co-owned by BMJ to the co-owners of the Journal, to publish the Work in this journal and any other BMJ products and to exploit all rights, as set out in our licence.

The Submitting Author accepts and understands that any supply made under these terms is made by BMJ to the Submitting Author unless you are acting as an employee on behalf of your employer or a postgraduate student of an affiliated institution which is paying any applicable article publishing charge ("APC") for Open Access articles. Where the Submitting Author wishes to make the Work available on an Open Access basis (and intends to pay the relevant APC), the terms of reuse of such Open Access shall be governed by a Creative Commons licence – details of these licences and which Creative Commons licence will apply to this Work are set out in our licence referred to above.

Other than as permitted in any relevant BMJ Author's Self Archiving Policies, I confirm this Work has not been accepted for publication elsewhere, is not being considered for publication elsewhere and does not duplicate material already published. I confirm all authors consent to publication of this Work and authorise the granting of this licence.

For peer review only

Interview guide

Experiences of stress-related exhaustion

Overall question:

-How do you experience being affected by stress-related exhaustion?

-Of falling ill?

-In what way has the experience of the illness had an impact on daily life?

Regarding work?

In contact with health care?

In relation to family, relatives and significant others?

Concerning yourself?

-What do you think is of importance for other people and health care professionals to know about this condition?

-Is there anything you would like to add in this interview?

Probing questions supporting narration

What do you mean by that?

How did that make you feel?

What do you think about that?

How did that affect you?

For peer review only

COREQ (COnsolidated criteria for REporting Qualitative research) Checklist

A checklist of items that should be included in reports of qualitative research. You must report the page number in your manuscript where you consider each of the items listed in this checklist. If you have not included this information, either revise your manuscript accordingly before submitting or note N/A.

Topic	Item No.	Guide Questions/Description	Reported on Page No.
**Domain 1: Research team and reflexivity**			
Personal characteristics			
Interviewer/facilitator	1	Which author/s conducted the interview or focus group?	11
Credentials	2	What were the researcher's credentials? E.g. PhD, MD	1
Occupation	3	What was their occupation at the time of the study?	1
Gender	4	Was the researcher male or female?	1
Experience and training	5	What experience or training did the researcher have?	1
Relationship with participants			
Relationship established	6	Was a relationship established prior to study commencement?	3
Participant knowledge of the interviewer	7	What did the participants know about the researcher? e.g. personal goals, reasons for doing the research	3
Interviewer characteristics	8	What characteristics were reported about the inter viewer/facilitator? e.g. Bias, assumptions, reasons and interests in the research topic	3
**Domain 2: Study design**			
Theoretical framework			
Methodological orientation and Theory	9	What methodological orientation was stated to underpin the study? e.g. grounded theory, discourse analysis, ethnography, phenomenology, content analysis	4
Participant selection			
Sampling	10	How were participants selected? e.g. purposive, convenience, consecutive, snowball	3
Method of approach	11	How were participants approached? e.g. face-to-face, telephone, mail, email	3
Sample size	12	How many participants were in the study?	3
Non-participation	13	How many people refused to participate or dropped out? Reasons?	3
Setting			
Setting of data collection	14	Where was the data collected? e.g. home, clinic, workplace	3
Presence of non-participants	15	Was anyone else present besides the participants and researchers?	3
Description of sample	16	What are the important characteristics of the sample? e.g. demographic data, date	3
Data collection			
Interview guide	17	Were questions, prompts, guides provided by the authors? Was it pilot tested?	3
Repeat interviews	18	Were repeat inter views carried out? If yes, how many?	3
Audio/visual recording	19	Did the research use audio or visual recording to collect the data?	3
Field notes	20	Were field notes made during and/or after the inter view or focus group?	3
Duration	21	What was the duration of the inter views or focus group?	3
Data saturation	22	Was data saturation discussed?	3
Transcripts returned	23	Were transcripts returned to participants for comment and/or	3

For peer review only

Topic	Item No.	Guide Questions/Description	Reported on Page No.
		correction?	
**Domain 3: analysis and findings**			
Data analysis			
Number of data coders	24	How many data coders coded the data?	4
Description of the coding tree	25	Did authors provide a description of the coding tree?	4
Derivation of themes	26	Were themes identified in advance or derived from the data?	4
Software	27	What software, if applicable, was used to manage the data?	4
Participant checking	28	Did participants provide feedback on the findings?	4
Reporting			
Quotations presented	29	Were participant quotations presented to illustrate the themes/findings? Was each quotation identified? e.g. participant number	5-9
Data and findings consistent	30	Was there consistency between the data presented and the findings?	9-10
Clarity of major themes	31	Were major themes clearly presented in the findings?	4-10
Clarity of minor themes	32	Is there a description of diverse cases or discussion of minor themes?	4-10

Developed from: Tong A, Sainsbury P, Craig J. Consolidated criteria for reporting qualitative research (COREQ): a 32-item checklist for interviews and focus groups. *International Journal for Quality in Health Care*. 2007. Volume 19, Number 6: pp. 349 – 357

Once you have completed this checklist, please save a copy and upload it as part of your submission. DO NOT include this checklist as part of the main manuscript document. It must be uploaded as a separate file.

For peer review only

Reporting checklist for qualitative study.

Based on the SRQR guidelines.

Instructions to authors

Complete this checklist by entering the page numbers from your manuscript where readers will find each of the items listed below.

Your article may not currently address all the items on the checklist. Please modify your text to include the missing information. If you are certain that an item does not apply, please write "n/a" and provide a short explanation.

Upload your completed checklist as an extra file when you submit to a journal.

In your methods section, say that you used the SRQRreporting guidelines, and cite them as:

O'Brien BC, Harris IB, Beckman TJ, Reed DA, Cook DA. Standards for reporting qualitative research: a synthesis of recommendations. Acad Med. 2014;89(9):1245-1251.

For peer review only

		Reporting Item	Page Number
**Title**			
	#1	Concise description of the nature and topic of the study identifying the study as qualitative or indicating the approach (e.g. ethnography, grounded theory) or data collection methods (e.g. interview, focus group) is recommended	1

Abstract

	#2	Summary of the key elements of the study using the abstract format of the intended publication; typically includes background, purpose, methods, results and conclusions	1

Introduction

Problem formulation	#3	Description and signifcance of the problem / phenomenon studied: review of relevant theory and empirical work; problem statement	1,2
Purpose or research question	#4	Purpose of the study and specific objectives or questions	3

Methods

Qualitative approach and research paradigm	#5	Qualitative approach (e.g. ethnography, grounded theory, case study, phenomenolgy, narrative research) and guiding theory if appropriate; identifying the research paradigm (e.g. postpositivist, constructivist / interpretivist) is also recommended; rationale. The rationale should briefly discuss the justification for choosing that theory, approach, method or technique rather than other options available; the assumptions and limitations implicit in those choices and how those choices influence study conclusions and transferability.	3

For peer review only

		As appropriate the rationale for several items might be discussed together.	
Researcher characteristics and reflexivity	#6	Researchers' characteristics that may influence the research, including personal attributes, qualifications / experience, relationship with participants, assumptions and / or presuppositions; potential or actual interaction between researchers' characteristics and the research questions, approach, methods, results and / or transferability	11
Context	#7	Setting / site and salient contextual factors; rationale	3
Sampling strategy	#8	How and why research participants, documents, or events were selected; criteria for deciding when no further sampling was necessary (e.g. sampling saturation); rationale	3
Ethical issues pertaining to human subjects	#9	Documentation of approval by an appropriate ethics review board and participant consent, or explanation for lack thereof; other confidentiality and data security issues	3
Data collection methods	#10	Types of data collected; details of data collection procedures including (as appropriate) start and stop dates of data collection and analysis, iterative process, triangulation of sources / methods, and modification of procedures in response to evolving study findings; rationale	3

For peer review only

Data collection instruments and technologies	#11	Description of instruments (e.g. interview guides, questionnaires) and devices (e.g. audio recorders) used for data collection; if / how the instruments(s) changed over the course of the study	3
Units of study	#12	Number and relevant characteristics of participants, documents, or events included in the study; level of participation (could be reported in results)	3
Data processing	#13	Methods for processing data prior to and during analysis, including transcription, data entry, data management and security, verification of data integrity, data coding, and anonymisation / deidentification of excerpts	3,4
Data analysis	#14	Process by which inferences, themes, etc. were identified and developed, including the researchers involved in data analysis; usually references a specific paradigm or approach; rationale	4,5
Techniques to enhance trustworthiness	#15	Techniques to enhance trustworthiness and credibility of data analysis (e.g. member checking, audit trail, triangulation); rationale	3

Results/findings

Syntheses and interpretation	#16	Main findings (e.g. interpretations, inferences, and themes); might include development of a theory or model, or integration with prior research or theory	4-10

For peer review only

Links to empirical data	#17	Evidence (e.g. quotes, field notes, text excerpts, photographs) to substantiate analytic findings	4-8
**Discussion**			
Intergration with prior work, implications, transferability and contribution(s) to the field	#18	Short summary of main findings; explanation of how findings and conclusions connect to, support, elaborate on, or challenge conclusions of earlier scholarship; discussion of scope of application / generalizability; identification of unique contributions(s) to scholarship in a discipline or field	9-10
Limitations	#19	Trustworthiness and limitations of findings	11
**Other**			
Conflicts of interest	#20	Potential sources of influence of perceived influence on study conduct and conclusions; how these were managed	11
Funding	#21	Sources of funding and other support; role of funders in data collection, interpretation and reporting	11

The SRQR checklist is distributed with permission of Wolters Kluwer © 2014 by the Association of American Medical Colleges. This checklist was completed on 02. March 2020 using https://www.goodreports.org/, a tool made by the EQUATOR Network in collaboration with Penelope.ai

For peer review only

BMJ Open

Facing a blind alley
Experiences of stress-related exhaustion – a qualitative study

Journal:	*BMJ Open*
Manuscript ID	bmjopen-2020-038230.R2
Article Type:	Original research
Date Submitted by the Author:	23-Jun-2020
Complete List of Authors:	Alsén, Sara; University of Gothenburg Institute of Health and Care Sciences, Sahlgrenska Academy<br>Ali, Lilas; Health and Care Sciences, Sahlgrenska academy<br>Ekman, Inger; Institute of Health and Care Sciences, Sahlgrenska Academy, University of Gothenburg; University of Gothenburg Centre for Person-Centred Care, Sahlgrenska Academy, University of Gothenburg<br>Fors, Andreas; Institute of Health of Care Sciences, Sahlgrenska Academy, University of Gothenburg; Gothenburg Centre for Person-Centred Care (GPCC),
**Primary Subject Heading**:	Mental health
Secondary Subject Heading:	Mental health, Qualitative research, Public health
Keywords:	PRIMARY CARE, MENTAL HEALTH, QUALITATIVE RESEARCH, PUBLIC HEALTH

SCHOLARONE™
Manuscripts

I, the Submitting Author has the right to grant and does grant on behalf of all authors of the Work (as defined in the below author licence), an exclusive licence and/or a non-exclusive licence for contributions from authors who are: i) UK Crown employees; ii) where BMJ has agreed a CC-BY licence shall apply, and/or iii) in accordance with the terms applicable for US Federal Government officers or employees acting as part of their official duties; on a worldwide, perpetual, irrevocable, royalty-free basis to BMJ Publishing Group Ltd ("BMJ") its licensees and where the relevant Journal is co-owned by BMJ to the co-owners of the Journal, to publish the Work in this journal and any other BMJ products and to exploit all rights, as set out in our licence.

The Submitting Author accepts and understands that any supply made under these terms is made by BMJ to the Submitting Author unless you are acting as an employee on behalf of your employer or a postgraduate student of an affiliated institution which is paying any applicable article publishing charge ("APC") for Open Access articles. Where the Submitting Author wishes to make the Work available on an Open Access basis (and intends to pay the relevant APC), the terms of reuse of such Open Access shall be governed by a Creative Commons licence – details of these licences and which Creative Commons licence will apply to this Work are set out in our licence referred to above.

Other than as permitted in any relevant BMJ Author's Self Archiving Policies, I confirm this Work has not been accepted for publication elsewhere, is not being considered for publication elsewhere and does not duplicate material already published. I confirm all authors consent to publication of this Work and authorise the granting of this licence.

For peer review only

Facing a blind alley

Experiences of stress-related exhaustion – a qualitative study

Sara Alsén[1,2], Lilas Ali[1,2,3], Inger Ekman[1,2], Andreas Fors[1,2,4]

[1] Institute of Health and Care Sciences, Sahlgrenska Academy, University of Gothenburg, Box 457, 405 30 Gothenburg, Sweden.

[2] Centre for Person-Centred Care (GPCC), University of Gothenburg, Sweden.

[3] Psychiatric department, Sahlgrenska University Hospital, Gothenburg, Sweden.

[4] Närhälsan Research and Development Primary Health Care, Region Västra Götaland, Sweden

Corresponding author:

Sara Alsén

Email: sara.alsen@gu.se.

Institute of Health and Care Sciences, Sahlgrenska Academy, University of Gothenburg, Sweden.

Box 457, 405 30 Gothenburg, Sweden.

For peer review only

Abstract

Introduction: Mental illness is a major concern in many countries. In Sweden, stress-related mental illness is currently the most frequent reason for sick leave.
Objective: This study aimed to explore patients´ experiences of stress-related exhaustion.
Design: A qualitative study with interview data analysed using a phenomenological hermeneutic method.
Setting: Participants were selected from public primary health care centres in a larger city in western Sweden.
Participants: Seven women and five men on sick leave from work due to stress-related exhaustion were included in the study.
Findings: Stress-related exhaustion was experienced as a loss of access to oneself and one's context and feelings of being trapped and lost in life. The condition had a significant impact on personal identity, raised existential issues, and was interpreted as facing a blind alley. Participants described following direction in life, being forced to stop, change direction, and act differently.
Conclusion: Stress-related exhaustion is a challenging, life-changing existential experience that involves a crisis with an opportunity for new insights. Careful consideration of patients´ narratives together with the expertise of health care professionals can be combined to improve health and optimise recovery based on individual´s situation.

For peer review only - http://bmjopen.bmj.com/site/about/guidelines.xhtml

Strengths and limitations of this study

- This study explored a less studied area, patient's experiences of stress-related exhaustion in the early stages of sick-leave from work.
- A sample of women and men of various ages was recruited to ensure representation.
- The finding gave insight into the meaning of living with stress-related exhaustion.
- The study included 12 participants' narratives, which could be argued do not apply to other people's lives in different contexts.

Introduction

Mental illness is a major concern in many countries,[1-4] and the days of work-related sick leave related to mental illness cause increased psychosocial stress.[5 6] In Sweden, the number of sick-leave days related to mental illness has more than doubled over the last decades. Stress-related mental illness is the most frequent reason for sick leave,[7 8] particularly in the human service sector.[9] Besides the negative impact on the patients´ well-being and risk of stigmatization,[10] its costs to society are high.[8] Research has found that exposure to stress increases the risk of both physical[11 12] and mental[13 14] symptoms, and cognitive impairment are frequently reported.[15] Distress has been shown to increase with unsupportive encounters.[16 17] Internationally, the term 'burnout' is used to describe the consequences of severe and long-term stress that is related to psychosocial factors at work.[11] The three key dimensions burnout are overwhelming exhaustion, feeling detached from work ('cynicism'), and diminishing efficiency at work. The definition of burnout has broadened beyond the core dimensions, and exhaustion and disengagement are also included along with non–work-related factors such as caregiver burden, work–family conflict, and unemployment.[18 19] Burnout, however, is not considered applicable in clinical practice.[20 21]

In 2003, the clinical diagnosis 'exhaustion disorder' was introduced in the Swedish version of the ICD-10 classification with the diagnostic code F43.8A to describe patients with stress-related exhaustion. The diagnostic criteria of at least one identified stressor, work- or non–work-related, should have been present for at least six weeks and that the clinical picture is dominated by a lack of psychological energy. Four of the following symptoms should be present almost every day for at least two weeks: concentration or memory impairment, emotional instability, reduced ability to cope with demands and/or time pressure, disturbed sleep, apparent physical weakness, and physical symptoms such as muscular pain.[22] Previous studies in patients with burnout have found that the condition may result from moral dilemmas and powerlessness in relation to work particularly in the context of health care.[23 24] The lead-in to burnout is characterised by symptoms such as headache, muscular pain, and fatigue and difficulties in living up to the emotional demands of family and friends. Self-image is threatened, and a need to refrain from social activities causes feelings of guilt.[25] Patients describe a situation in which their ideal self is not matched by reality.[26 27] Underlying factors

For peer review only - http://bmjopen.bmj.com/site/about/guidelines.xhtml

of stress-related exhaustion are mainly associated with work demand, but also with relational factors in private life or a lack of work/life balance.[28 29 30]

Previous research has mainly focused on the triggers for burnout or addressed specific occupational groups such as health care professionals. However, stress-related exhaustion is increasing in other demographic groups, and an improved understanding of how the condition affects everyday life of patients on sick leave is important to reduce suffering and enhance recovery. This study aimed to explore patients' experiences of stress-related exhaustion during absence from work on sick leave.

Method

Design

An explorative qualitative interview study, the data were analysed using phenomenological hermeneutics.[31] This approach is designed to develop deeper understanding of individuals' perspectives by interpreting their narratives as texts. The present study is based on the Standards for Reporting Qualitative Research (SRQR) guidelines.[32]

Participants and setting

Participants were selected from public primary health care centres in a large city in the western Sweden. Designated health care professionals screened patients on sick leave due to stress related exhaustion from medical records. After patients consent the first author contacted the patients by telephone to schedule a time for an interview. A representative sample was recruited to ensure a variety of men and women of various ages. Participants were invited to participate based on the following inclusion criteria: (a) diagnosis exhaustion disorder by a physician and (b) ongoing sick leave no longer than six months due to exhaustion disorder (c), having a physical and mental capacity to participate in an interview. The interviews were conducted until the authors considered the research question as fully answered. Nineteen people were contacted and informed about the study; three declined, and four withdrew before the interview due to lack of sufficient energy to participate. In total, 12 people were recruited, 7 women and 5 men, aged 25 to 46 years (Table 1). Six of the participants were married and six were single, and their current percentage sick leave hours ranged between 50-100%, except for one who resumed full time work two days before the interview.

Data collection

The interviews were conducted between June 2018 and February 2019 by the first author. Each participant decided whether their interview took place at the first author's office, at a café, or in the participant's home. The interviews were performed 2 to 4 months after sick leave had commenced and lasted between 28 to 79 minutes (median 45 minutes) and were recorded and transcribed verbatim by the first author. The aim of the study was explained to the participants, and they were asked to narrate their experiences. The opening question was 'How do you experience being affected by stress-related exhaustion?'. To obtain more in-depth narratives of the experiences, the interviewer asked probing questions such as, 'What does that mean to you? How do you feel about that?'

For peer review only - http://bmjopen.bmj.com/site/about/guidelines.xhtml

Patient and Public Involvement

The study was conducted within the Gothenburg University, Centre for Person-Centred Care (GPCC), where patient and public involvement is essential. A patient representative was involved in the present project, which conducted research in the area of stress-related mental illness.

Ethical considerations

Approval for the study was obtained from the Ethical Review Board at the University of Gothenburg (Dnr: 497-17, T 526-18), and the study conformed to the Declaration of Helsinki. The participants received verbal and written information about the study and assurance that they could withdraw at any time. All interview data were treated confidentially. Sharing lived experiences can be difficult and may raise sensitive issues. If required, patients were offered consultation with a counsellor. Written consent was obtained from each participant.

Table 1 Characteristics of the participants

Gender	Age
1: Male	38
2: Female	30
3: Female	25
4: Female	29
5: Female	37
6: Female	43
7: Female	43
8: Male	46
9: Female	36
10: Male	28
11: Male	41
12: Male	25

Data analysis

The interviews were analysed using phenomenological hermeneutical method. The intention with this method is to generate an understanding of the meaning of a particular phenomenon, in this case, living with stress-related exhaustion. The method is inspired by Ricoeur´s theory of interpretation [31 33], which has been used in numerous studies during the last 20 years.[34 35] The analysis consists of three intertwined steps: naïve reading, structural analysis, and interpretation of the whole.[31] In the naïve reading, the text is read repeatedly to grasp its meaning as a whole. In the structural analysis, the text is divided into meaning units, which are abstracted and formed into subthemes. The structural analysis entails a dialectical movement shifting focus between the meaning units and the overall impression of the text. Several structural analyses and interpretations of the text were performed in this study. In the final part of the analysis; interpretation of the whole, the entire text was re-read and interpreted in relation to the authors´ pre-understandings, the naïve reading, the structural analyses, research related to the area and Jaspers philosophy[36] on limit situation to formulate a comprehensive understanding of the combined narratives.[31]

For peer review only - http://bmjopen.bmj.com/site/about/guidelines.xhtml

Findings

The first impression of the text revealed that experiencing stress-related exhaustion had a significant impact on the participants and their everyday lives. Paralysing fatigue, in combination with cognitive impairment, contributed to difficulties in taking part and engaging in life. This raised feelings of disability and powerlessness, which contributed to participants' experiences of losing their foothold in life. Thoughts about life´s meaning arose, as did feelings of being lost and trapped. Life was perceived as indefinitely paused, with possibilities of change limited, no matter how much they struggled.

Structured analysis

Table 2 Subthemes, themes, and main theme

Main theme	Theme	Subtheme	Example of quotes for each theme
Facing a blind alley	Loss of access to oneself	Constraints determine conditions	"I am confused so my family has helped me keep track of time, appointments, and, helped me pay the bills, and so on" (10)
		Loss of self-recognition	
		Deprivation of dignity	
	Endless struggle	Managing everyday life	"I live alone with a child who has a disability. I never sleep a whole night and have not done that in nine years, which leads to consequences. Here I am. I keep on doing everything without seeing any way out of this" (6).
		Searching for explanations and understanding	
		Process of negotiation	
	Life on hold	Lost in the middle of nowhere	"My life is paused. It is on hold. Life goes on out there. My friends and other people's lives go on, but mine is in some limbo because nothing is going on. I exist, but there is no life in it. My studies are interrupted. Everything is interrupted, and I do not live. Living dead or…" (12)
		Excluded from life	
		Rethinking life	

<u>Loss of access to oneself</u>

Experiencing stress-related exhaustion made participants feel they had lost access to themselves. This loss was expressed in three subthemes: *constraints determine conditions*, *loss of self- recognition*, and *deprivation of dignity* (Table 2).

Constraints determine conditions

The participants lost access to functions they previously had in everyday life. The obstacles they faced took over their abilities to act as they wished in life. Health problems such as pain, anxiety, and sleep disturbance were common, and paralysing fatigue usually forced them to rest. The loss of vital functions made every chore a challenge, and these limitations contributed to feelings of disability and regression; sometimes even their orientation in time and space was affected.

> I am confused, so my family has helped me keep track of time, appointments, helped me pay the bills, and so on (10).

Participants also described having difficulty holding a conversation because they lacked energy and had problems finding the right words.

Loss of self-recognition

For peer review only - http://bmjopen.bmj.com/site/about/guidelines.xhtml

Feeling a loss of control due to the constraints determining their everyday conditions contributed to a loss of self-recognition. Participants described their previous selves as able to manage a high pace or to handle several tasks at the same time – things that were no longer possible. The participants experienced a significant discrepancy between their previous and their actual selves, and this loss seemed to affect their personality and felt as if parts of themselves were missing.

> I have been able to work from four-thirty in the morning until nine p.m. After work, I arrived home and continued with household chores, for example, washing, cleaning, and other things. I used to have such a high capacity (8).

This experience caused feelings of frustration and anger, and feelings of shame and guilt towards their significant others. The noticeable difference in their appearance worried both themselves and people close to them.

> Many asked how I felt. I replied as you usually do: "I am tired". And there was a colleague who especially pushed me to seek help because I was not myself (10).

Deprivation of dignity

Their reduced abilities to perform activities that had previously given them pride and self-esteem, contributed to participants' experiences of losing dignity. Previous roles and positions, both private and professional, changed with the condition, and their earlier strength and courage decreased.

> I was sitting in the waiting room, feeling week, helpless, and useless. We justify our existence by being productive in some way, and one feels bad when one is not able to pay one's dues and needs to take up the physician's time. I felt worthless (12).

Deprivation of dignity was especially evident with health care professionals, being considered a patient and treated as a sick person. Having stress-related exhaustion was an overwhelming experience, and not being cared for and treated with respect made the participants feel undignified.

> I lost my foothold and felt very weak when the physician expressed himself that way: 'If you do not plan to kill yourself, I don't need to take you on sick leave.' It was terrible! I have never identified myself as a person who gives up like that (5).

<u>Endless struggle</u>

The exemplars revealed that the participants experienced an endless struggle, which was expressed in the following three subthemes: *managing everyday life*, *searching for explanations and understanding*, and *process of negotiation* (Table 2).

Managing everyday life

It was a challenging struggle to maintain the tempo necessary for coping with daily life. Life offered limited opportunities for change, and the participants saw no other options than to continue their struggle. They used strategies and compensated for their disabilities by

For peer review only - http://bmjopen.bmj.com/site/about/guidelines.xhtml

reducing commitments both at work and in private life. Trying to act as they had before required a great effort, but they felt obliged to their family to do so.

> I live alone with a child who has a disability. I never sleep a whole night and have not done that in nine years, which leads to consequences. Here I am. I keep on doing everything without seeing any way out of this (6).

Searching for explanations and understanding

The participants searched for explanations and understanding about their condition and they struggled to find answers

> I always try to reach answers to why this happened to me, but I do not know if I have reached something yet. Everything is just speculation, and I hope something is right, but I don't know (12).

They were often referred between different physicians and health care facilities, which contributed to their increased frustration and stress. Repeated visits and answers that nothing was wrong, despite their feeling ill, left them feeling they were a burden, and they started to distrust their ability to understand themselves

> In the end, I started thinking, Have I become mad? Have I misunderstood my signals? Should my life be like this for the rest of my life? (9)

Process of negotiation

Various answers from health care professionals were under constant negotiation and involved participants rejecting, defending, and trying to accept their condition.

> I experienced a big conspiracy against me when everyone had this mental approach, and I was sure that it was biological. All the bodily symptoms made me believe something physically was wrong with me. Then four doctors, who all had this mental approach, made me feel overpowered, and I had to let go of the hypothesis I'd hung onto (12).

Accepting their limitations and the fact that they had been affected by stress-related exhaustion posed challenges, especially if their treatment by health care professionals was less supportive and involved challenging process of negotiation with themselves.

> The most difficult thing for me was to admit I was sick. It took me several days, or it took several weeks, actually. It was tough, but once I had admitted it to myself, had emailed my boss, had sent the message to the private healthcare insurance, then I released that burden and accepted the situation (8).

Lost in the middle of nowhere

The narratives also showed that participants felt lost in the middle of nowhere, which was expressed in the following three subthemes: *life on hold*, *excluded from life*, and *rethinking life* (Table 2).

For peer review only - http://bmjopen.bmj.com/site/about/guidelines.xhtml

Life on hold

Other people's lives seemed to move ahead, leaving the participants behind, and they felt they were going around in circles with little ability to influence the situation. They felt emptiness, meaninglessness, and a life with restricted content.

> My life is paused. It is on hold. Life goes on out there. My friends and other people's lives go on, but mine is in some limbo because nothing is going on. I exist, but there is no life in it. My studies are interrupted. Everything is interrupted, and I do not live. Living dead or… (12)

The fact that no time interval for recovery could be specified raised feelings of uncertainty and powerlessness. These feelings were a struggle to handle when searching for motivation to continue the everyday fight. The advice from health care professionals was to rest, which was perceived as pointless because it had no noticeable effect on their recovery.

> I do not know what needs to happen or what to do to get better. I am resting all the time, but I notice no improvement. It is like trying to start a car without a battery. Pointless. The motor is broken, so it is no idea. You turn the key, but it is dead. So, I honestly do not know how to manage it or how someone should succeed in doing so (11).

Excluded from life

The participants felt excluded from essential parts of their lives such as work and spending time with family and friends. They described feeling excluded from their familiar, everyday context and felt uncertain about what the future would hold.

> I have somehow been excluded from my old life. It is as if I look at my past life through my memories. It is like a great villa. In this villa is my early life, but the door is locked, and I stand looking into a window and see everything I could do before. None of this I can do anymore. It doesn't work (12).

Activities that previously gave life its meaning were no longer possible, which contributed to limiting the joyful elements in their everyday lives. Spending time on things that previously gave life its meaning led instead to grief and sadness.

> ... He asked me if I had thoughts of killing myself, which I never reflected on before. However, when she asked me, I noticed that I had been thinking about it. Because it was the football World Cup and I couldn't watch TV, I couldn't eat, I couldn't do anything joyful (8).

Rethinking life

Having stress-related exhaustion made the participants reflect on their previous and present life situations. They re-assessed previous actions and goals in life, and their prior way of living was questioned.

For peer review only - http://bmjopen.bmj.com/site/about/guidelines.xhtml

> I have hated lying on the couch all my life, and now it's the only thing I can do. It is such a rethinking of life. That you, in the end, are committed and obliged to do what you have never wanted to do. It's like the body says you don't get away with this, or there is no shortcut (12).

Most of the participants felt responsible for what had happened. An motional burden arose when they realised that their condition affected themselves and others. Now when facing the consequences they felt disappointment to themselves and to others. Looking back could raise feelings of guilt and shame, but also insights into what needed to change in their lives.

> For me, I see this basically as something positive. It does not feel positive, but I think it is a life lesson not to act as I have done so far. So, actually, the illness is a consequence of how I have lived my life, and the onset of the illness is a turning point, thus a chance to turn it over (3).

Interpretation of the whole

Having stress-related exhaustion was a life-changing experience for the participants, and had a significant impact on their self-conception and their everyday lives. Their entire existence changed, and their selves and lives were perceived as unrecognisable. They struggled to regain access to themselves and a sense of meaning. This situation was interpreted as facing a blind alley, taking a fruitless and mistaken direction in life that forced them to stop, change direction, and act differently. The blind alley, however, can represent both a crisis and an opening to new insights.

Discussion

In contrast to other studies,[37 38] the participants in our study were interviewed in the early stages of their sick-leave. Our findings that participants struggled with a loss of vital functions, questioned their prior ways of life, and faced a new life situation. This is consistent with findings from a study later in the rehabilitation phase,[37] and thus indicates that the condition has a great impact on people in both early and later stages of sick leave. Another study after several months of rehabilitation showed that life was experienced as living in darkness and struggling with the creation of meaning.[38] This indicate that symptom burden and challenges described in the present study may continue long term.

We interpreted a link between stress-related exhaustion to people's personal identities and existential challenges. The experience of *losing access to oneself* and to one's context affects the persons' entire view of themselves. 'The engine is broken, and it is impossible to start the car', as one participant expressed. The discrepancy between previous and present capacities contributed to *loss of self-recognition* and was linked to one's identity. The participants struggled not only with the challenge of understanding their condition, but also with their need to understand themselves and their identities. This is in line with findings from previous studies, which have described the condition as a struggle with a threatening nothingness,[39] being disconnected from oneself,[17] and a disconnection from the body and the world.[37]

For peer review only - http://bmjopen.bmj.com/site/about/guidelines.xhtml

The impact of stress-related exhaustion on a person´s identity also raises questions about life and its meaning. An understanding on the meaning of being affected by stress-related exhaustion can be understood as a *limit situation*, as described by the existential philosopher and psychiatrist Karl Jaspers (1883–1969). Limit situations are those such as death, struggling, suffering, guilt, and existential anxiety that remain unchanged. People do not perceive the limit situation until they confront it concretely and personally. Limit situations are like a wall we run into and then collapse, and we cannot avoid confronting them.[36] The main theme of our analysis, *facing a blind alley,* can be both as a limit situation and a metaphor for how stress-related exhaustion affects a person's existence and raises existential challenges such as 'Who am I without my professional identity?' 'Who am I without the ability to perform?' and 'What is valuable in my life when I cannot live life as desired?' A further interpretation is that the participants experienced *life on hold* and were *excluded from life* because relevance and meaning were missing. They were powerless in a situation they could not overcome, life became meaningless. This can be understood as expressing an existential feeling of unrelatedness. Such existential vulnerability is reminiscent of Jasper's metaphor of the broken mussel shell.[40] Throughout life, humans create sustainable foundations through their views of life, like building a house that creates conditions and protects against unpredictable experiences. In a limit situation, this sustainable foundation is threatened, and the person, much like a mussel without a shell, is homeless and unprotected from life's challenges.[40](p 248)

Consistent with our findings, Jasper's metaphor points out the vulnerability of people who struggle to understand and explain their experience of stress-related exhaustion to themselves and others. This vulnerability, combined with a lack of respectful care and treatment from health care professionals, has a negative impact on the person's identity and self. Engelbrektsen et al. describe how patients who seek care for stress-related exhaustion experience the encounter with the general practitioner as a battle and feel they are distrusted by others. This results in disconnection from their lifeworld, which in turn triggers shame reactions.[17] In our study, this experience was interpreted as *deprivation of dignity* due both to the guilt and shame caused by their views of their condition and feeling unheard in their encounters with health care professionals. Shame has been described as an emotion of self-assessment that causes the person to feel anxiety at the thought of how he or she is seen and judged by others. Unsupportive encounters affect how the persons attribute values of themselves as human beings with subsequently increased distress.[16] Knapstaden et al. found that shame among people on sick leave might prolong absence.[41] The participants thus struggled to understand and explain their condition not only to themselves but also to others in light of their feelings of loss of self and context. Ricoeur distinguishes between two fundamental aspects of the self or identity,[42] which he calls *ipse* (selfhood) and *idem* (sameness). Ipse is that kind of inexpressible inner core that marks the core of who we are. Idem, on the other hand, is a more external way of identifying the self. Ipse identifies 'who' the self is and idem is 'what' the self consists of. [42] Our study findings indicate health care professionals focus on idem (sameness) and disregard ipse (selfhood), which means they may not meet the person beyond the disorder. This raises concerns, especially as the

For peer review only - http://bmjopen.bmj.com/site/about/guidelines.xhtml

condition has a significant impact on the person's identity. Hence, it is essential to know the person with stress-related exhaustion as a person first, as emphasised in person-centred care. Person-centred care takes the patient´s narratives as the starting point for patients and health care professionals to co-create individual plans of care and shared decision making.[43] Carefully listening to patients´ narratives is a core component to identify his/her resources and needs, and to achieve a common understanding. Considering patients´ narratives in the clinical encounter enables them to become an active part in finding solutions for health problems and strengthen their self-care abilities.[43 44]

Jaspers concept is useful to understand stress-related exhaustion as a limit situation, which is an experience of importance to humans, a life event that one cannot avoid.[36] Facing a blind alley implies that the participants are in a limit situation; they are forced to stop, rethink their decisions, and find a new direction and a more suitable way in life. Jaspers' limit situation also entails an opening to deeper insights, and therefore carries an inherent contradiction: one can experience and understand both one's limitations and one's possibilities. When the inner and outer contexts the participants built up collapse and a new direction in life is necessary, they start *rethinking life*. Earlier life choices and life goals are re-assessed; they question their life situation, identity, value, and meaning of life. *Rethinking life* seems to be the starting point to regain both a way forward in life and the belief that life is meaningful.

<u>Methodological limitations</u>

The study included 12 participants' narratives, which could be argued do not to apply to other people's lives. The qualitative approach, however, aims not to reach generalizable findings, but to attain a deeper understanding of people's experiences; this study can, therefore, provide an understanding of people's lived experiences of stress-related exhaustion. The phenomenological hermeneutic approach aims to obtain understanding of people's individual perspectives via the interpretation of their narratives as texts.[31 33] To ensure trustworthiness, the qualitative framework for interpretative research by de Witt & Ploeg's[45] was applied, and which comprises five characteristics: balanced integration, openness, concreteness, resonance, and actualization. *Balanced integration* was achieved by using a phenomenological hermeneutical approach where quotes from the participants were related to Karl Jaspers concept of limit-situation. Openness was demonstrated by accounting for decisions that were made throughout the study, such as inclusion criteria, cessation of data collection and the analysis process. A reflexive process established the preconceived attitudes and beliefs to reduce biases in the process of data collection, analysis and interpretation. Regarding concreteness, the findings were presented by examples of quotes that situate the reader concretely in the experience and contexts of being affected by stress-related exhaustion. Resonance was reproduced by the intensity and reflective nature of the participant's voice in their expression of the condition. For actualization, the study results were not conclusive but could be interpreted by readers to deepen their understanding.

For peer review only - http://bmjopen.bmj.com/site/about/guidelines.xhtml

Conclusion and clinical implications

This study revealed that stress-related exhaustion is a challenging and life-changing experience that encompasses both a crisis and an opening towards new insights. When encountering patients with stress-related exhaustion, it is of importance to acknowledge the condition as severe and complex, which affects the person's identity and includes existential issues. Persons affected by stress-related exhaustion are often in urgent need of a caring dialogue to deal with feelings of shame, guilt and meaninglessness. Healthcare professionals are responsible for initiating this dialogue and for creating a respectful care environment that allows patients to narrate about their experiences and existential issues, which is a key to understanding the condition and finding new opportunities in their situation. Thoroughly listening to the patient´s narrative enables to acquire knowledge about the patient, as a person, with needs, including wishes and feelings. This, together with the expertise of the healthcare professional, can be used to co-create a personal health plan, also including realistic and achievable goals, to be followed up during recovery. One crucial aspect is also to identify and use the patient´s resources and competences to make it possible for the person to see the crisis as a way to learn from it. Such a holistic approach, which take into account the personal experience of the condition, may avoid deprivation of the person´s dignity, and can be implemented by applying person-centred care in clinical practice. Hence, we suggest combining the patients´ and the health care professionals´ perspectives in the clinical encounter, as a way to improve health and to optimize recovery for people with stress-related exhaustion.

Contributions: All authors participated in the design of the study. SA conducted and transcribed the interviews. SA performed the analysis with critical input from LA, IE, and AF. SA drafted the manuscript, which was reviewed and edited by LA, IE, and AF. All authors approved the final manuscript. The authors wish to acknowledge the participants for sharing their experiences.

Funding: This study was financed by grants from The Swedish Research Council for Health, Working Life and Welfare (reference number 2016-07418, 2017-00557 and 2019-01726) and from the Swedish state under the ALF agreement between the Swedish government and the country (ALFGBG-772191 and ALFGBG-932659). This work was also supported by The University of Gothenburg Centre for Person-centred Care (GPCC), Sweden. GPCC is funded by the Swedish Government's grant for Strategic Research Areas (Care Sciences) and the University of Gothenburg, Sweden. The funders had no role in the design of the study, data collection, analysis, or interpretation.

Competing interests: None declared.

Ethics approval: Regional Ethical Review Board of the University of Gothenburg.

Data sharing statement: No additional data are available.

For peer review only - http://bmjopen.bmj.com/site/about/guidelines.xhtml

Word count: 4,598 (exclusive of abstract, tables, and references)

References

1. WHO. Depression and other common mental disorders: Global health estimates. Geneva: World Health Organisation. Available at: http://www.who.int/mental_health/management/depression/prevalence_global_health_estimat es/en. 2017
2. Henderson M, Harvey SB, Øverland S, et al. Work and common psychiatric disorders. 2011;104:198-207. doi: 10.1258/jrsm.2011.100231
3. Siegrist J. Chronic psychosocial stress at work and risk of depression: evidence from prospective studies. *European Archives of Psychiatry and Clinical Neuroscience* 2008;258(Supplement 5):115-19. doi: 10.1007/s00406-008-5024-0
4. EU-OSHA. European Agency for Safety and Health at Work (2014). Calculating the costs of workrelated stress and psychosocial risks – A literature review. Luxembourg: Publications Office of the European Union. Avalable at: https://osha.europa.eu/en/publications/calculating-cost-work-related-stress-and-psychosocial-risks.
5. Bültmann JHU, Huibers PGMM, Van Amelsvoort VL, et al. Psychological Distress, Fatigue and Long-Term Sickness Absence: Prospective Results From the Maastricht Cohort Study. *Journal of Occupational and Environmental Medicine* 2005;47(9):941-47. doi: 10.1097/01.jom.0000172865.07397.9a
6. Leka S, Jain A, Iavicoli S. An Evaluation of the Policy Context on Psychosocial Risks and Mental Health in the Workplace in the European Union: Achievements, Challenges, and the Future. *BioMed Research International* 2015;2015 doi: 10.1155/2015/213089
7. Försäkringskassan. Försäkringskassan [Swedish Social Insurance Agency]. Uppföljning av sjukfrånvarons utveckling 2019. 2019:https://www.forsakringskassan.se/wps/wcm/connect/fb32bcf5-7ed4-4356-b18b-6adc7ca20fcd/1573-19-svar-regeringsuppdrag.pdf?MOD=AJPERES&CVID=.
8. OECD. Mental Health and Work: Sweden. Paris OECD Publishing, 2013. Available at: https://read.oecd-ilibrary.org/employment/mental-health-and-work-sweden_9789264188730-en#page1.
9. Stenbeck M, Persson G. Chapter 10: Working life, work environment and health. *Scandinavian Journal of Public Health* 2006;34(67_suppl):229-45. doi: 10.1080/14034950600677295
10. Verhaeghe M, Bracke P. Associative Stigma among Mental Health Professionals: Implications for Professional and Service User Well-Being. *Journal Of Health And Social Behavior* 2012;53(1):17-32. doi: 10.1177/0022146512439453
11. Melamed S, Shirom A, Toker S, et al. Burnout and Risk of Cardiovascular Disease: Evidence, Possible Causal Paths, and Promising Research Directions. *Psychological Bulletin* 2006;132(3):327-53. doi: 10.1037/0033-2909.132.3.327
12. Chandola T. Chronic stress at work and the metabolic syndrome: prospective study.(WORKPLACE HEALTH & BENEFITS), 2006:3.
13. Clark C, Pike C, McManus S, et al. The contribution of work and non-work stressors to common mental disorders in the 2007 Adult Psychiatric Morbidity Survey. *Psychological Medicine* 2012;42(4):829-42. doi: 10.1017/S0033291711001759
14. Cole DC, Ibrahim S, Shannon HS, et al. Work and life stressors and psychological distress in the Canadian working population: a structural equation modelling approach to analysis of the 1994 National Population Health Survey. *Chronic diseases in Canada* 2002;23(3):91.
15. Jonsdottir IH, Nordlund A, Ellbin S, et al. Cognitive impairment in patients with stress-related exhaustion. *Stress* 2013;16(2):181-90. doi: 10.3109/10253890.2012.708950

For peer review only - http://bmjopen.bmj.com/site/about/guidelines.xhtml

16. Dolezal L. Shame, Vulnerability and Belonging: Reconsidering Sartre's Account of Shame. *Human Studies* 2017;40(3):421-38. doi: 10.1007/s10746-017-9427-7
17. Engebretsen KM, Bjorbækmo WS. Naked in the eyes of the public: A phenomenological study of the lived experience of suffering from burnout while waiting for recognition to be ill. *Journal of Evaluation in Clinical Practice* 2019 doi: 10.1111/jep.13244
18. Daalen G, Willemsen T, Sanders K, et al. Emotional exhaustion and mental health problems among employees doing "people work": the impact of job demands, job resources and family-to-work conflict. *International Archives of Occupational and Environmental Health* 2009;82(3):291-303. doi: 10.1007/s00420-008-0334-0
19. Lindström C, Aman J, Norberg AL. Parental burnout in relation to sociodemographic, psychosocial and personality factors as well as disease duration and glycaemic control in children with Type 1 diabetes mellitus. *Acta paediatrica (Oslo, Norway : 1992)* 2011;100(7):1011. doi: 10.1111/j.1651-2227.2011.02198.x
20. Maslach C, Schaufeli WB, Leiter MP. JOB BURNOUT. *Annual Review of Psychology* 2001;52(1):397-422. doi: 10.1146/annurev.psych.52.1.397
21. Shirom A, Melamed S. A Comparison of the Construct Validity of Two Burnout Measures in Two Groups of Professionals. *International Journal of Stress Management* 2006;13(2):176-200. doi: 10.1037/1072-5245.13.2.176
22. Grossi G, Perski A, Osika W, et al. Stress-related exhaustion disorder – clinical manifestation of burnout? A review of assessment methods, sleep impairments, cognitive disturbances, and neuro-biological and physiological changes in clinical burnout. *Scandinavian Journal of Psychology* 2015;56(6):626-36. doi: 10.1111/sjop.12251
23. Severinsson E. Moral stress and burnout: Qualitative content analysis. *Nursing & Health Sciences* 2003;5(1):59-66. doi: 10.1046/j.1442-2018.2003.00135.x
24. Billeter-Koponen S, Fredén L. Long-term stress, burnout and patient–nurse relations: qualitative interview study about nurses' experiences. *Scandinavian Journal of Caring Sciences* 2005;19(1):20-27. doi: 10.1111/j.1471-6712.2005.00318.x
25. Ekstedt M, Fagerberg I. Lived experiences of the time preceding burnout. *Journal Of Advanced Nursing* 2005;49(1):59-67. doi: 10.1111/j.1365-2648.2004.03264.x
26. Gustafsson G, Norberg A, Strandberg G. Meanings of becoming and being burnout – phenomenological-hermeneutic interpretation of female healthcare personnel's narratives. *Scandinavian Journal of Caring Sciences* 2008;22(4):520-28. doi: 10.1111/j.1471-6712.2007.00559.x
27. Arvidsdotter T, Marklund B, Kylen S, et al. Understanding persons with psychological distress in primary health care. *Scandinavian Journal Of Caring Sciences, 2016, Vol 30, Iss 4* 2016;30(4)
28. Hasselberg K, Jonsdottir IH, Ellbin S, et al. Self-reported stressors among patients with Exhaustion Disorder: An exploratory study of patient records. *Acta Veterinaria Scandinavica* 2014;14(1):66. doi: 10.1186/1471-244X-14-66
29. Sandmark H, Renstig M. Understanding long-term sick leave in female white-collar workers with burnout andstress-related diagnoses: a qualitative study. *Bmc Public Health* 2010;10(1):Article Number: 210. doi: 10.1186/1471-2458-10-210
30. Håkansson C, Ahlborg G. Occupational imbalance and the role of perceived stress in predicting stress-related disorders. *Scandinavian Journal of Occupational Therapy* 2018;25(4):278-87. doi: 10.1080/11038128.2017.1298666
31. Lindseth A, Norberg A. A phenomenological hermeneutical method for researching lived experience. *Scandinavian Journal of Caring Sciences* 2004;18(2):145-53. doi: 10.1111/j.1471-6712.2004.00258.x
32. O'brien CB, Harris BI, Beckman JT, et al. Standards for Reporting Qualitative Research: A Synthesis of Recommendations. *Academic Medicine* 2014;89(9):1245-51. doi: 10.1097/ACM.0000000000000388

For peer review only - http://bmjopen.bmj.com/site/about/guidelines.xhtml

33. Ricœur P. Interpretation theory : discourse and the surplus of meaning: Fort Worth, Tex. Texas Christian Univ. Press 1976.
34. Baxter R, Sandman P-O, Björk S, et al. Illuminating Meanings of Thriving for Persons Living in Nursing Homes. *The Gerontologist* 2019 doi: 10.1093/geront/gnz142
35. Fors A, Dudas K, Ekman I. Life is lived forwards and understood backwards - Experiences of being affected by acute coronary syndrome: A narrative analysis.(Author abstract). *International Journal of Nursing Studies* 2014;51(3):430.
36. Jasper K. (1994) Limit situations. In Karl Jaspers Basic philosophical writings (Erlich E. ELHPGB, eds), Humanities press, New Jersey, pp. 96-104.
37. Jingrot M, Rosberg S. Gradual Loss of Homelikeness in Exhaustion Disorder. *Qualitative Health Research* 2008;18(11):1511-23. doi: 10.1177/1049732308325536
38. Arman M, Hammarqvist AS, Rehnsfeldt A. Burnout as an existential deficiency - lived experiences of burnout sufferers. *Scandinavian Journal of Caring Sciences* 2011;25(2):294-302. doi: 10.1111/j.1471-6712.2010.00825.x
39. Rehnsfeldt A, Arman M. A pilgrimage on the road to understanding of life in experiences of cancer and burnout syndrome. *Scandinavian Journal of Caring Sciences* 2008;22(2):275-83. doi: 10.1111/j.1471-6712.2007.00531.x
40. Jaspers KPdWBVvJS. Psychologie der Weltanschauungen. Berlin: Verlag von Julius Springer.
41. Knapstad M, Øverland S, Henderson M, et al. Shame among long-term sickness absentees: Correlates and impact on subsequent sickness absence. *Scandinavian Journal of Public Health* 2014;42(1):96-103. doi: 10.1177/1403494813500590
42. Ricœur P. Oneself as another. New ed. ed. Chigago: Chigago : University of Chicago Press 1994.
43. Ekman I, Swedberg K, Taft C, et al. Person-Centered Care — Ready for Prime Time. *European Journal of Cardiovascular Nursing* 2011;10(4):248-51. doi: 10.1016/j.ejcnurse.2011.06.008
44. Leplege A, Gzil F, Cammelli M, et al. Person-centredness: Conceptual and historical perspectives. *Disability and Rehabilitation: Person-centred Rehabilitation: Rhetoric or Reality?* 2007;29(20-21):1555-65. doi: 10.1080/09638280701618661
45. De Witt L, Ploeg J. Critical appraisal of rigour in interpretive phenomenological nursing research. *Journal of Advanced Nursing* 2006;55(2):215-29. doi: 10.1111/j.1365-2648.2006.03898.x

For peer review only

For peer review only - http://bmjopen.bmj.com/site/about/guidelines.xhtml

COREQ (COnsolidated criteria for REporting Qualitative research) Checklist

A checklist of items that should be included in reports of qualitative research. You must report the page number in your manuscript where you consider each of the items listed in this checklist. If you have not included this information, either revise your manuscript accordingly before submitting or note N/A.

Topic	Item No.	Guide Questions/Description	Reported on Page No.
**Domain 1: Research team and reflexivity**			
Personal characteristics			
Interviewer/facilitator	1	Which author/s conducted the interview or focus group?	11
Credentials	2	What were the researcher's credentials? E.g. PhD, MD	1
Occupation	3	What was their occupation at the time of the study?	1
Gender	4	Was the researcher male or female?	1
Experience and training	5	What experience or training did the researcher have?	1
Relationship with participants			
Relationship established	6	Was a relationship established prior to study commencement?	3
Participant knowledge of the interviewer	7	What did the participants know about the researcher? e.g. personal goals, reasons for doing the research	3
Interviewer characteristics	8	What characteristics were reported about the inter viewer/facilitator? e.g. Bias, assumptions, reasons and interests in the research topic	3
**Domain 2: Study design**			
Theoretical framework			
Methodological orientation and Theory	9	What methodological orientation was stated to underpin the study? e.g. grounded theory, discourse analysis, ethnography, phenomenology, content analysis	4
Participant selection			
Sampling	10	How were participants selected? e.g. purposive, convenience, consecutive, snowball	3
Method of approach	11	How were participants approached? e.g. face-to-face, telephone, mail, email	3
Sample size	12	How many participants were in the study?	3
Non-participation	13	How many people refused to participate or dropped out? Reasons?	3
Setting			
Setting of data collection	14	Where was the data collected? e.g. home, clinic, workplace	3
Presence of non-participants	15	Was anyone else present besides the participants and researchers?	3
Description of sample	16	What are the important characteristics of the sample? e.g. demographic data, date	3
Data collection			
Interview guide	17	Were questions, prompts, guides provided by the authors? Was it pilot tested?	3
Repeat interviews	18	Were repeat inter views carried out? If yes, how many?	3
Audio/visual recording	19	Did the research use audio or visual recording to collect the data?	3
Field notes	20	Were field notes made during and/or after the inter view or focus group?	3
Duration	21	What was the duration of the inter views or focus group?	3
Data saturation	22	Was data saturation discussed?	3
Transcripts returned	23	Were transcripts returned to participants for comment and/or	3

For peer review only

For peer review only - http://bmjopen.bmj.com/site/about/guidelines.xhtml

Topic	Item No.	Guide Questions/Description	Reported on Page No.
		correction?	
**Domain 3: analysis and findings**			
Data analysis			
Number of data coders	24	How many data coders coded the data?	4
Description of the coding tree	25	Did authors provide a description of the coding tree?	4
Derivation of themes	26	Were themes identified in advance or derived from the data?	4
Software	27	What software, if applicable, was used to manage the data?	4
Participant checking	28	Did participants provide feedback on the findings?	4
Reporting			
Quotations presented	29	Were participant quotations presented to illustrate the themes/findings? Was each quotation identified? e.g. participant number	5-9
Data and findings consistent	30	Was there consistency between the data presented and the findings?	9-10
Clarity of major themes	31	Were major themes clearly presented in the findings?	4-10
Clarity of minor themes	32	Is there a description of diverse cases or discussion of minor themes?	4-10

Developed from: Tong A, Sainsbury P, Craig J. Consolidated criteria for reporting qualitative research (COREQ): a 32-item checklist for interviews and focus groups. *International Journal for Quality in Health Care*. 2007. Volume 19, Number 6: pp. 349 – 357

Once you have completed this checklist, please save a copy and upload it as part of your submission. DO NOT include this checklist as part of the main manuscript document. It must be uploaded as a separate file.

For peer review only

Reporting checklist for qualitative study.

Based on the SRQR guidelines.

Instructions to authors

Complete this checklist by entering the page numbers from your manuscript where readers will find each of the items listed below.

Your article may not currently address all the items on the checklist. Please modify your text to include the missing information. If you are certain that an item does not apply, please write "n/a" and provide a short explanation.

Upload your completed checklist as an extra file when you submit to a journal.

In your methods section, say that you used the SRQRreporting guidelines, and cite them as:

O'Brien BC, Harris IB, Beckman TJ, Reed DA, Cook DA. Standards for reporting qualitative research: a synthesis of recommendations. Acad Med. 2014;89(9):1245-1251.

		Reporting Item	Page Number
**Title**			
	#1	Concise description of the nature and topic of the study identifying the study as qualitative or indicating the approach (e.g. ethnography, grounded theory) or data collection methods (e.g. interview, focus group) is recommended	1

For peer review only

Abstract

	#2	Summary of the key elements of the study using the abstract format of the intended publication; typically includes background, purpose, methods, results and conclusions	1

Introduction

Problem formulation	#3	Description and signifcance of the problem / phenomenon studied: review of relevant theory and empirical work; problem statement	1,2
Purpose or research question	#4	Purpose of the study and specific objectives or questions	3

Methods

Qualitative approach and research paradigm	#5	Qualitative approach (e.g. ethnography, grounded theory, case study, phenomenolgy, narrative research) and guiding theory if appropriate; identifying the research paradigm (e.g. postpositivist, constructivist / interpretivist) is also recommended; rationale. The rationale should briefly discuss the justification for choosing that theory, approach, method or technique rather than other options available; the assumptions and limitations implicit in those choices and how those choices influence study conclusions and transferability.	3

For peer review only

		As appropriate the rationale for several items might be discussed together.	
Researcher characteristics and reflexivity	#6	Researchers' characteristics that may influence the research, including personal attributes, qualifications / experience, relationship with participants, assumptions and / or presuppositions; potential or actual interaction between researchers' characteristics and the research questions, approach, methods, results and / or transferability	11
Context	#7	Setting / site and salient contextual factors; rationale	3
Sampling strategy	#8	How and why research participants, documents, or events were selected; criteria for deciding when no further sampling was necessary (e.g. sampling saturation); rationale	3
Ethical issues pertaining to human subjects	#9	Documentation of approval by an appropriate ethics review board and participant consent, or explanation for lack thereof; other confidentiality and data security issues	3
Data collection methods	#10	Types of data collected; details of data collection procedures including (as appropriate) start and stop dates of data collection and analysis, iterative process, triangulation of sources / methods, and modification of procedures in response to evolving study findings; rationale	3

For peer review only

Data collection instruments and technologies	#11	Description of instruments (e.g. interview guides, questionnaires) and devices (e.g. audio recorders) used for data collection; if / how the instruments(s) changed over the course of the study	3
Units of study	#12	Number and relevant characteristics of participants, documents, or events included in the study; level of participation (could be reported in results)	3
Data processing	#13	Methods for processing data prior to and during analysis, including transcription, data entry, data management and security, verification of data integrity, data coding, and anonymisation / deidentification of excerpts	3,4
Data analysis	#14	Process by which inferences, themes, etc. were identified and developed, including the researchers involved in data analysis; usually references a specific paradigm or approach; rationale	4,5
Techniques to enhance trustworthiness	#15	Techniques to enhance trustworthiness and credibility of data analysis (e.g. member checking, audit trail, triangulation); rationale	3

Results/findings

Syntheses and interpretation	#16	Main findings (e.g. interpretations, inferences, and themes); might include development of a theory or model, or integration with prior research or theory	4-10

For peer review only

Links to empirical data	#17	Evidence (e.g. quotes, field notes, text excerpts, photographs) to substantiate analytic findings	4-8
**Discussion**			
Intergration with prior work, implications, transferability and contribution(s) to the field	#18	Short summary of main findings; explanation of how findings and conclusions connect to, support, elaborate on, or challenge conclusions of earlier scholarship; discussion of scope of application / generalizability; identification of unique contributions(s) to scholarship in a discipline or field	9-10
Limitations	#19	Trustworthiness and limitations of findings	11
**Other**			
Conflicts of interest	#20	Potential sources of influence of perceived influence on study conduct and conclusions; how these were managed	11
Funding	#21	Sources of funding and other support; role of funders in data collection, interpretation and reporting	11

The SRQR checklist is distributed with permission of Wolters Kluwer © 2014 by the Association of American Medical Colleges. This checklist was completed on 02. March 2020 using https://www.goodreports.org/, a tool made by the EQUATOR Network in collaboration with Penelope.ai

For peer review only
